# Automated four-dimensional long term imaging enables single cell tracking within organotypic brain slices to study neurodevelopment and degeneration

**DOI:** 10.1038/s42003-019-0411-9

**Published:** 2019-05-01

**Authors:** Jeremy W. Linsley, Atmiyata Tripathi, Irina Epstein, Galina Schmunk, Elliot Mount, Matthew Campioni, Viral Oza, Mariya Barch, Ashkan Javaherian, Tomasz J. Nowakowski, Siddharth Samsi, Steven Finkbeiner

**Affiliations:** 1 Gladstone Center for Systems and Therapeutics, San Francisco, CA 94158 USA; 2 Department of Anatomy, University of California, San Francisco, CA 94158 USA; 3 Luxembourg Centre for Systems Biomedicine, University of Luxembourg, Luxembourg, L-4367 Luxembourg; 4 Neuroscience Graduate Program, University of California, San Francisco, CA 94158 USA; 5 Biomedical Sciences and Neuroscience Graduate Program, University of California, San Francisco, CA 94143 USA; 6 Taube/Koret Center for Neurodegenerative Disease, Gladstone Institutes, San Francisco, CA 94158 USA; 7 Department of Neurology, University of California, San Francisco, CA 94158 USA; 8 Department of Physiology, University of California, San Francisco, CA 94158 USA; 9 Present Address: MIT Lincoln Laboratory, Lexington, MA 02421 USA

**Keywords:** Time-lapse imaging, Neurodegeneration, Confocal microscopy, Animal disease models, Tissue culture

## Abstract

Current approaches for dynamic profiling of single cells rely on dissociated cultures, which lack important biological features existing in tissues. Organotypic slice cultures preserve aspects of structural and synaptic organisation within the brain and are amenable to microscopy, but established techniques are not well adapted for high throughput or longitudinal single cell analysis. Here we developed a custom-built, automated confocal imaging platform, with improved organotypic slice culture and maintenance. The approach enables fully automated image acquisition and four-dimensional tracking of morphological changes within individual cells in organotypic cultures from rodent and human primary tissues for at least 3 weeks. To validate this system, we analysed neurons expressing a disease-associated version of huntingtin (HTT586Q138-EGFP), and observed that they displayed hallmarks of Huntington’s disease and died sooner than controls. By facilitating longitudinal single-cell analyses of neuronal physiology, our system bridges scales necessary to attain statistical power to detect developmental and disease phenotypes.

## Abstract (web summary)

Jeremy Linsley, Atmiyata Tripathi et al. optimise culturing techniques and develop a microscopy platform and that can automatically image and track single neurons within organotypic slices for up to a three-week period. Using a model of polyQ neurodegeneration, they show that this method can assess longitudinal cell changes with high accuracy.

## Introduction

Deciphering the changes that occur within the brain that underlie its function, development, and pathophysiology is a central problem in neuroscience. Because developmental and neurological disease-relevant phenotypes often develop over protracted periods of time and are frequently confined to specific cell types, it can be difficult to determine the significance of incremental changes from static snapshots. Most temporally resolved approaches for identifying cellular phenotypes within live tissue also have limited scalability, confining the ability to power analyses of incremental changes within the heterogeneity and complexity of the brain, and restricting the capability to determine whether phenotypic perturbations have long-lasting consequences. To overcome these issues, automated longitudinal imaging (ALI) studies provide an ideal platform to generate temporally resolved datasets in cell culture, and have often challenged the prevailing models of the aetiology of neurodegenerative diseases. For example, ALI revealed that inclusion bodies (IBs), a pathological hallmark of Huntington’s disease (HD), are part of a beneficial coping response rather than the causative factor or incidental factor in HD neurodegeneration^1,2^. Additionally, the ability to track individual neurons over time in culture facilitates single-cell analysis and is 100–1000 times more sensitive than population-based studies that rely on a single snapshot in time^3^.

Nevertheless, ALI has previously been limited to imaging 2-dimensional (2D) monolayer cultures of disassociated cells, which lack many of the important biological features of 3-dimensional (3D) brain tissue. Key events during brain development such as cell divisions, differentiations and migrations occur in 3D space over extended periods of time, and have not previously been accessible to ALI^4–6^. Furthermore, although 2D ALI analysis of Parkinson’s disease (PD)^7,8^, amyotrophic lateral sclerosis (ALS)^2,9,10^, and HD^1,11^ cell models have yielded valuable insights into the time course of pathology of neurodegenerative disease, it has also become clear that the relationship of each neuron to the cells around it is critical to the pathophysiology of these diseases. For instance, our knowledge of the role of non-neuronal cells and their 3D relationship to neurons within the brain during neurodegeneration is continually being expanded^12–16^. Recent evidence has also begun to accumulate to support the propagation hypothesis, which posits that pathogenic proteins may be transmitted from cell to cell throughout the brain in HD, synucleinopathies, ALS, and Alzheimer’s disease (AD) in a prion-like fashion^17–19^. As many neurological diseases are strongly linked to abnormal neural circuitry, the necessity of a longitudinal four-dimensional (4D) understanding of the time course of activity of individual neurons has become critical to understanding the dysfunction of neurons in both health and disease^20–22^.

Organotypic slice culture (OSC) is an appealing alternative approach to dissociated cell culture that preserves much of the 3D architecture and connectivity of tissue in vivo^23–26^, while maintaining genetic tractability and allowing accessibility of neurons to microscopy. OSC enables simultaneous visualisation of different cell types, including neurons, neuronal precursors, microglia and astrocytes in a defined circuitry. Moreover, OSC can be prepared from primary human tissue and murine models of any genetic background, including models that show reduced postnatal survival. As a result, OSC has become increasingly popular as a platform for modelling neurodegenerative diseases, such as HD^27,28^, AD^29–32^, and PD^33,34^, and neurodevelopmental perturbations such as viral infections^35^.

Furthermore, because assays in tissue slices in culture can be scaled up for high-throughput drug screens^25,36^, it is a promising approach for generating treatments for disease. Despite these advantages, previous longitudinal imaging approaches of OSC have been constrained because tissue had to be transported out of the incubator and onto a microscope stage that was not temperature controlled, limiting the throughput and imaging period. Additionally, re-identification of the region of interest within a given tissue was performed manually, making analysis cumbersome and potentially introducing human bias^37,38^.

Here we report a method for time-lapse live 4D imaging of cells within OSC and its application to the study of longitudinal changes in both neurodegenerative disease and brain development. We custom built and programmed an integrated automated spinning disc confocal microscope capable of reproducibly returning to precise 3D positions within OSC over longitudinal imaging periods, enabling high throughput identification, tracking, and analysis of single cells in tissue. Using an improved, imaging-optimised culturing setup, we extended the ability to track individual neurons in human OSC for up to 3 weeks, and tracked changes in cell number, velocity, morphology, position, and neuronal health on a protracted scale. Additionally, we modelled HD in OSC and found that transfection with DNA encoding a version of an N-terminal fragment of the huntingtin protein that causes Huntington’s disease (HTT586Q138-EGFP) affected morphology, IB formation, and decreased survival of neurons in culture. Using our innovative high throughput platform, studies tracking cellular phenotypes over protracted time-frames can be performed at resolution and scales necessary to deconvolve the sequential events underlying cellular and molecular changes during neurodegeneration and development with high spatiotemporal precision.

## Results

### Automated 4D time lapse imaging of OSC

Longitudinal analysis of single neurons in primary tissues or in vitro-derived organoids^39^ requires automated imaging solutions to continually return to the same location in 3D space. To accomplish this, we custom-built a fully-automated high throughput imaging system capable of longitudinal single cell analysis in slices (Supplementary Fig. 1a, Supplementary Movie 1). It incorporates a technology we developed previously for imaging two-dimensional cultures in which a fiduciary mark on the bottom of the plate is used in combination with custom software to allow repeated automated return to the same location on the imaging plate, even if the plate has been removed from the stage in the interim^1^. To maximise throughput while retaining high spatial resolution, we built the system with a spinning disk confocal and integrated its automated control into the system with Micromanager^40^. An automated three axis stage was added to an inverted microscope base to automate *z*-axis movements and optical sectioning. To enable return to the same starting *z*-height before each imaging session, we integrated the perfect focus system (PFS), which uses reflected light from near-IR LED to maintain focus at a pre-set distance above the bottom of the plate. Once the initial *z*-plane is determined, the automated microscope disengages the focus lock and moves in programmable *z*-steps to each imaging plane, giving a consistent depth of imaging at each time point with high precision in three dimensions. Additionally, a robotic arm was integrated to move imaging plates from an automated incubator to the microscope stage and back before and after imaging, resulting in full automation of the entire process. The spinning disc confocal, working in combination with two sCMOS cameras, and a six line laser launch facilitates fast, simultaneous two-colour high content imaging. These features make our custom-built microscope uniquely adaptable to studies of single cells in 3D as well as through programmable time-lapse (4D). Studies that require video rate image acquisition can be integrated over the course of several time points to provide a wide range of imaging parameters capable of capturing subtype and long-term changes in cellular dynamics (Supplementary Fig. 1C).

**Fig. 1:**
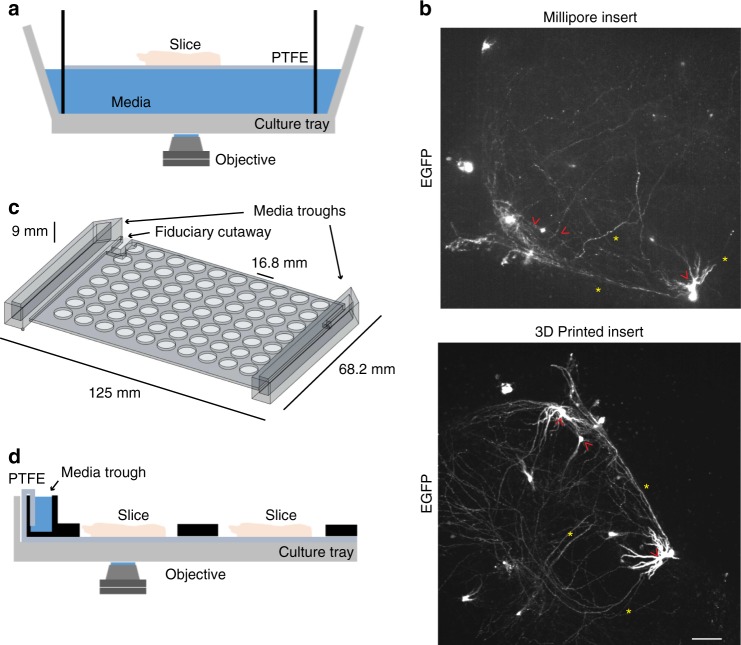
Optimisation of slice culture inserts for improved and high-throughput imaging organotypic tissue. **a** Cutaway illustration of a commercially available cell-culture insert (Millicell) in which a slice is suspended on a polytetrafluoroethylene (PTFE) membrane at the interface of the medium (blue) and air within a well of a culture tray. The inverted microscope objective images through medium to slice. **b** Comparison of representative confocal images of EGFP expression within transfected hippocampal slices imaged using Millicell insert (top) and 3D printed insert (bottom). Red arrowheads indicate comparable somata and yellow asterisks indicate neurites. Scale = 50 μm. **c** Illustration of the design of a 3D printed, full plate insert with attached medium troughs (arrows) sized to fit into standard cell culture tray. **d** Cutaway illustration of customised 3D printed array insert containing medium troughs (black objects) within a cell-culture tray, with reduced working distance of the microscope objective to the slice. PTFE membrane stretches along the length of the culture tray and wicks media from media trough to slice samples (pink objects)

Long-term OSC requires tissue to be kept at an interface of air and culture media. We initially performed pilot experiments using commercially available slice culture inserts which use a semipermeable membrane and a reservoir of medium below to maintain the slice at the interface between medium and air (Fig. 1a). Although our microscope was able to capture images of EGFP transfected slices cultured on commercial inserts (Fig. 1b), we encountered several technical problems that limited their use in long-term automated imaging. First, the insert sagged over time, making tracking of individual neurons within the slice difficult as their location in space progressively changed along each axis. Second, when the insert was placed in an imaging plate compatible with an automated stage for multiplexed imaging of multiple slices, it was not anchored to the plate and would frequently move when moving the plate. Finally, placing the medium beneath the slice interferes with imaging by the inverted microscope as it extends the working distance from the objective to the slice, increasing the amount of scattered light and placing greater demands on the optics to generate high quality images.

To address these limitations, we redesigned the culturing setup by fabricating an insert that allows simultaneous culturing of up to 70 individual slices at a consistent and reduced working distance from an inverted microscope objective (Fig. 1c, d). The insert was 3D printed with acrylonitrile-butadiene-styrene (ABS) material and customised to fit inside a standard, commercially available optical cell-culture tray. It uses standard well spacing typical of commercially available 96-well optical plates compatible with high-throughput imaging (see methods section). Slices are placed on top of polytetrafluoroethylene (PTFE) membrane, which lines the tray and connects with the medium troughs on the sides of the insert (Fig. 1d). Capillary action wicks the medium to flow towards the centre of the plate, wetting the membrane and providing the liquid interface for slices. The slices are centred on the membrane exposed by the holes of the insert, allowing free access to air (Fig. 1d). Changing the medium in the troughs every 1–2 days, maintains the slice culture with minimal disruption to the position of the slice. The long-term neuronal health of P7 mouse hippocampal slices as assessed by gross cellular morphology was comparable between a commercially available insert and slices on the 3D printed insert (Supplementary Fig. 2). The working distance from the objective to the top of the slice was reduced by around 1 mm, greatly enhancing the quality of fluorescence imaging of EGFP-transfected neurons within the slice. By decreasing light scattering and increasing signal, this new system enables a substantial spatial resolution improvement over conventional inserts, for both somata and processes of transfected neurons in brain slices (Fig. 1b).

### Automated, confocal 4D tracking of neurons within live cultures

**Fig. 2:**
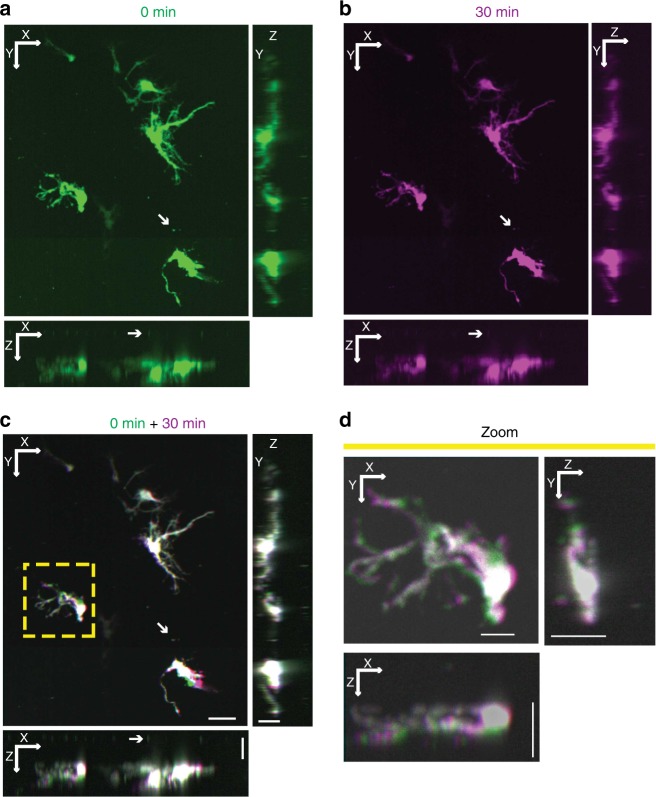
Automated 4D imaging of single neurons within organotypic slice culture. **a** Maximum projections of representative hippocampal slice culture transfected with EGFP along *X*–*Y* (top left), *X*–*Z* (bottom left) and *Y*–*Z* (top right) axes from automated confocal imaging with 184 *z*-slices. Arrow points to fluorescent beads placed between the transfected slice and the PTFE membrane. **b** A second confocal image stack of the same EGFP-transfected hippocampal slice culture taken after 30 min and projected into along *X*–*Y* (top left), *X*–*Z* (bottom left) and *Y*–*Z* (top right) axes. **c** Alignment of 0-min (green) and 30-min (purple) 3D projections from **a** and **b** to create a 4D image projected along *X*–*Y* (top left), *X*–*Z* (bottom left) and *Y*–*Z* (top right) axes. White areas indicate regions of overlap between time points, while areas of purple or green indicate regions of neuronal movement over time. *XY* scale = 50 μm, *YZ* and *XZ* scale = 20 μm. **d** Magnification of projections along *X*–*Y* (top left), *X*–*Z* (bottom left) and *Y*–*Z* (top right) axes from yellow box in **c** showing subtle movements (areas of purple and green) over time within a single neuron in 4D. *XY* Scale = 15 μm, *YZ* and *XZ* scale = 20 μm

To demonstrate the ability to image in 3D and 4D, we prepared organotypic slices of primary mouse hippocampus from P7 mice. To visualise single cells, we transfected them with pGW1-CMV-EGFP^1^ expressed from a CMV enhancer promoter in the pGW1 plasmid using a biolistic gene gun after 5 days in culture, which results in a high proportion of neurons among the labelled cells (Supplementary Fig. 3). Slices were placed on the 3D printed insert with fluorescent beads pre-coated on the PTFE membrane. Automated 0.5-μm-spaced *z*-stacks of images were collected, and a 3D reconstruction of the slice was generated (Fig. 2a). The 3D reconstruction showed that beads on the surface of the slice could easily be resolved in the *Z*-dimension from neurons within the slice, and the 3D morphology of transfected neurons could be visualised (Fig. 2a). Computational deconvolution can be used to increase the resolution in the *z*-axis (Supplementary Fig. 3). Additional automated imaging of the same field of view was performed using the fiducial mark and PFS to allow a second 3D reconstruction of transfected neurons in the slice and an aligned 4D overlay of images from the two time points (Fig. 2b-d). Using the 4D approach within a brain slice and a voxel size of 0.3225 μm × 0. 3225 μm × 0.5 μm, changes could be monitored within a single field of view in as little as 4 min, or as few as 9 min if imaging the area of an entire slice. In pilot experiments, small changes in morphology could be observed over time with 9 min intervals that became more pronounced across multiple intervals. Using an arbitrary 30-min imaging interval, we observed distinct and pronounced changes in neuronal morphology within our slice (Fig. 2c, d). These data demonstrate that our system is capable of 4D ALI and of resolving single cell morphological changes over time.

**Fig. 3:**
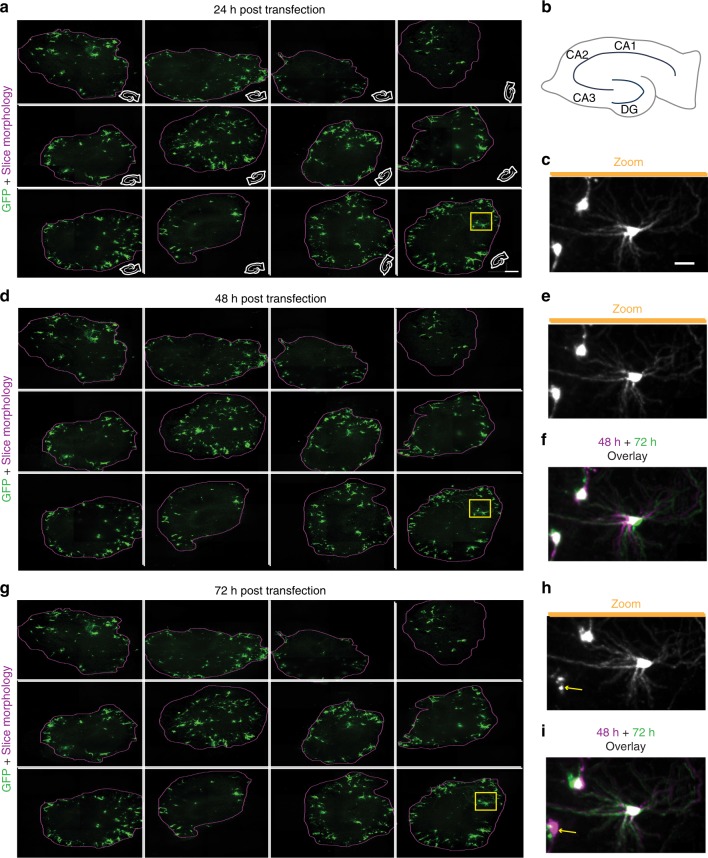
Longitudinal automated high-content, multiplexed imaging to detect changes within single neurons. **a** Maximum z-projections of 12 hippocampal slices transfected with EGFP and imaged with automation at high resolution 24 h post transfection (hpt) (left, scale bar 300 µm). Purple line represents edge of the slice. White cartoon inset shows the orientation of slice within the well. **b** Schematic of a hippocampal slice with locations of cornu ammonis (CA) 1, CA2, CA3, and dentate gyrus (DG) subregions. Miniaturised versions of same schematic were used in insets of **a**. **c** Magnification of three individual neurons within yellow box in **a** demonstrating high content of imaging approach. Scale = 50 μm. **d** Maximum *z* projections of the same 12 slices from **a** 48 hpt. **e** Expansion of same neurons within yellow box in **c** 48 hpt. **f** Overlay of imaging from 24 (magenta, same as **c**) and 48 hpt (green) showing differences in morphology over time. **g** Maximum *z*-projections of the same 12 slices from **a** and **d** 72 hpt. **h** Expanded view of yellow box in **g**. **i** Overlay of imaging 48 hpt (magenta, same as **e**) and 72 hpt (green) showing differences in morphology over time. Arrows indicate death of a neuron at 72 hpt that is present at 48 hpt

Throughput is often limited in longitudinal imaging of tissue as samples are commonly processed serially over time. However, parallelising longitudinal imaging of multiple samples at once reduces the microscope time usage, improves dynamic control of imaging and sample conditions, and facilitates imaging of multiple slices from a single brain or anatomically similar slices from multiple brains for time-matched comparisons. We next sought to increase the throughput of the automated system in order to image multiple slices from a single brain in parallel. Using hippocampal slices from P7 mice transfected with EGFP, automated imaging was performed on 12 slices within an imaging plate, each with 3 × 3 arrays to capture the entire slice area, and 100 *z* steps of 1 μm to capture the slice volume at each time point. The high-content of the sCMOS camera enabled imaging of individual neuronal processes within the slice with high definition (Fig. 3a), and the system collected 3240 images in around 30 min. Images were stitched together, maximum projected, and aligned per time point to allow tracking of individual neurons and their processes within the slices (Fig. 3a–i). Alignment of neurons across time points facilitated observations of morphology changes, movements, and neuronal death (Fig. 3g–i) within each slice across time points. Thus, the automated 4D imaging of slice cultures can be scaled to make it possible to image multiple slices rapidly in parallel, increasing throughput and the robustness of the analysis by enabling matched comparisons of neurons in multiple slices across a single brain.

### Dynamic changes of neuronal morphology in long-term microscopy

**Fig. 4:**
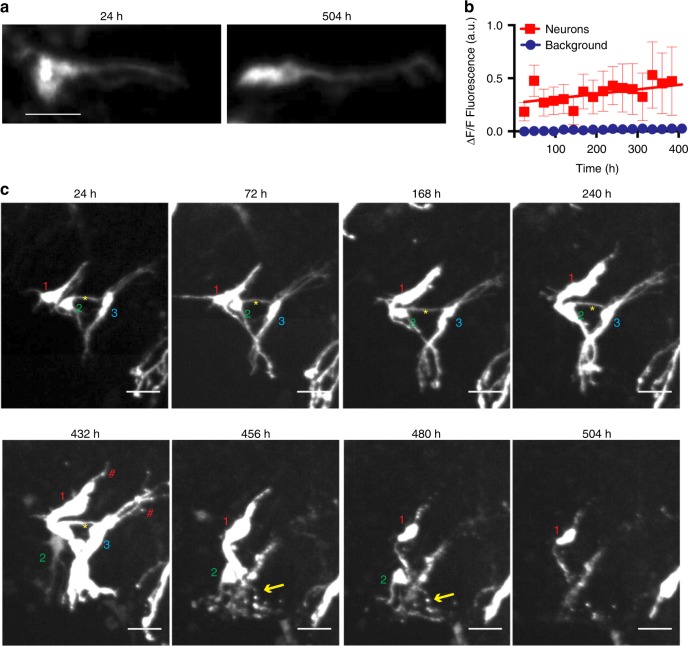
Complex changes in morphology preceding neuronal death revealed by longitudinal tracking of single cells in organotypic slice culture over weeks. **a** Maximum *z*-projections of a single EGFP-transfected neuron within a hippocampal slice culture imaged at 24 h post-transfection (hpt) (left) and 504 hpt (right) showing consistent EGFP signal. Scale = 40 μm. **b** Quantification of EGFP signal from single neurons (*n* = 12) imaged over 384 h showing increased EGFP signal over time. *R*^2^ = 0.8 SEMs are indicated. **c** Time-lapse time course of three neurons over 504 h showing changing morphology and neuronal death. Each neuron in a volume is labelled with a number (1–3) at the beginning of the experiment. When a cell dies, the software notes the event and the label disappears from the image. Yellow asterisks indicate apparent contacts between EGFP-labelled neurons. Red hashtags indicate areas of blebbing in unhealthy neurons. Yellow arrows indicate apparent neurite degeneration. Scale = 50 μm

With the ability to repeatedly return to the same 3D space over time, we can extend the time course of neuronal imaging for as long as a slice survives in culture, facilitating analyses of morphology changes and neuronal death over that time (Supplementary Movie 2). Fluorescent protein expression is stable over the lifetime of cultured neurons^41^, and the abrupt loss of fluorescent protein signal was shown previously to mark neuronal death^1^ by indicating the timepoint at which rupture of the plasma membrane occurred. Nevertheless, stability of EGFP over weeks in slice culture after biolistic gene gun transfection has not been assayed. We found persistent and stable EGFP fluorescence for up to 504 h of imaging (Fig. 4a). Furthermore, time lapse quantification of EGFP expression within tracked neurons over the course of 504 h showed that EGFP expression gradually, but significantly increased (0.7 a.u./h) compared to background intensity (*p* < 0.0001 ANCOVA) (Fig. 4b). Thus, the reproducible temporal expression profile of EGFP made it suitable to track neuronal morphology and death in slice culture over long periods.

Long-term tracking of individual neurons over 480 h revealed complex dynamics among individual neurons within a slice (Supplementary Movie 3). In a representative slice (Fig. 4c), by 24 h post-transfection (hpt), neuronal processes emerged and became more defined with extensive branching and apparent contacts between neurons. By 432 hpt, clumping of EGFP was visible in cells, indicative of unhealthy cultures. By 456 hpt, two neurons start to look fragmented and display degeneration of neurites, similar to degeneration phenotypes observed in neuronal culture^8^. Furthermore, contacts between neurons were retracted, and one neuron appeared to lose membrane integrity as its EGFP signal faded. By 504 hpt, only one neuron remained, and its fluorescence intensity was diminished, suggesting loss of membrane integrity and imminent death. Interestingly, while the somata of most neurons usually remained relatively stationary within the slice throughout their lifetime, others could be seen moving *en passant* between stationary neurons through the tissue (Fig. 4c). Somata movement was rare, and only 1% of all neurons tracked across 24 slices (7 of 622) moved more than two somata lengths during imaging. In each case, neuronal somata were stationary for multiple days of imaging before moving (Fig. 4c). Thus, with the use of our system, the interplay among neurons before degeneration can be analysed within 3D tissue over several weeks for the first time, to our knowledge.

### Longitudinal tracking of primary human neural stem cells in 4D imaging

**Fig. 5:**
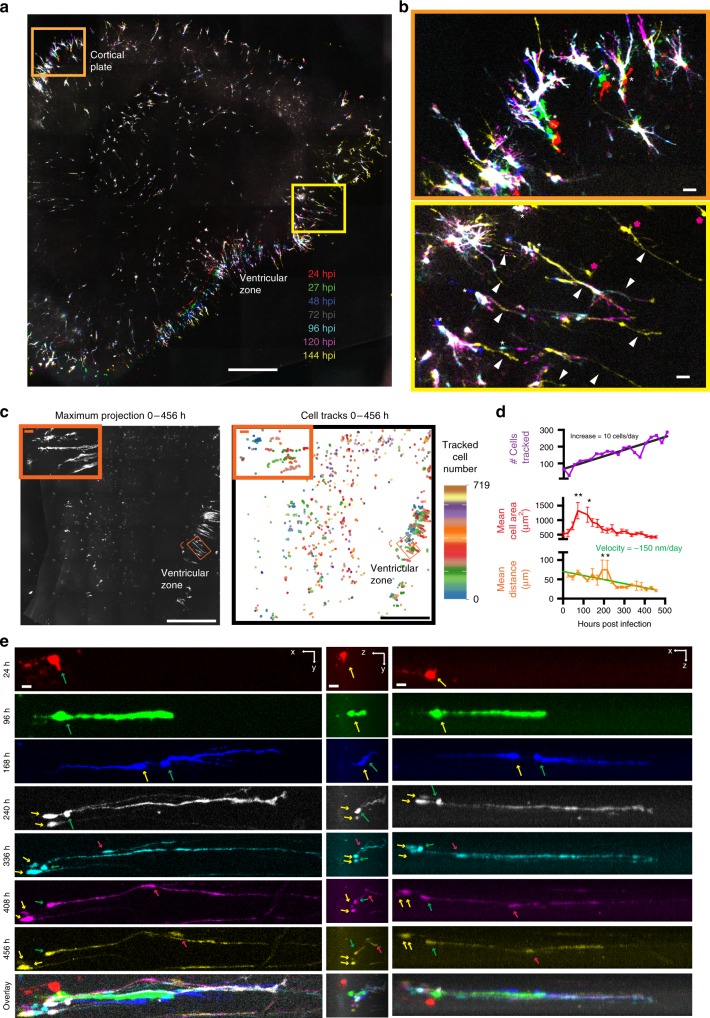
Longitudinal imaging and tracking of single cells in human neocortical slice culture for extended time periods. **a** Maximum projections of longitudinal imaging of a representative GW19 human cortical slice expressing EGFP at 24, 27, 48, 72, 96, 120 and 144 hpi overlayed into a single image to show RG cell movement. Scale = 1 mm. **b** Zoom in of orange (top, cortical plate) and yellow (bottom, ventricular zone) boxes from **a** showing variable cell movement (white asterisks), process extensions (white arrowheads), and newly labelled cells (pink asterisks) over time across the slice. Scale = 100 µm. **c** (Left) Maximum projection of a representative GW22 slice across all Z and time series covering 0-468 hpi with 24 h intervals. (Left Inset) Zoom in of orange box showing region of furthest movement. Curved orange arrow indicates direction of 48° rotation to produce orange zoom in box. (Right) Automated cell tracking traces of the centroid from 719 segmented cells from left image overlayed across 469 h time course with each cell labelled in a distinct hue showing changes in its position over time. (Right Inset) Zoom in of orange box showing tracks of furthest movement. Curved orange arrow indicates direction of 48° rotation to produce orange zoom in box. Scale = 1 mm. **d** (Top) Quantification of number of cells segmented and tracked over time. Black line represents linear regression from which a rate of 10 cells per day was derived. (Middle) Quantification of mean cell area and (Bottom) mean distance of all cells tracked in **c** (*R*^2^ = 0.82, ANOVA Tukey multiple comparisons **p < 0.01, *p < 0.05). Green line represents linear regression of mean distance travelled from which an average velocity of −150 nm per day was derived (*R*^2^ = 0.6, ANOVA Tukey multiple comparisons **p < 0.01, *p < 0.05). Error bars show SD. **e** Longitudinal time series of an oRG cell from **c** (green arrow) undergoing divisions into daughter cells (yellow arrows), and serving as a scaffold for a migrating cell (red arrow) in *x*–*y* from a single *z* slice (left panels, scale = 40 μm), *z*–*y* brightest point projection (middle panels, scale = 30μm), and *x*–*z* brightest point projection (right panels, scale = 40 μm) over the course of 432 h of imaging

Although murine models have been instrumental for studying the principles of brain development, human developing cerebral cortex exhibits a greatly expanded pool of neural stem cells, as well as cell types and neurodevelopmental events not commonly found in mouse^6,42^. Given the protracted developmental timescales in human, strategies for maintaining and imaging human primary cells are urgently needed, and we next sought to test whether our improved slice culture setup could be amenable to imaging organotypic slices of human tissues. We fabricated a new 3D printed insert with 15 large wells and cultured tissue slices of primary human brain tissue at gestational week 19 (GW19), which are substantially larger than mouse hippocampal slices (Supplementary Fig. 4). To follow dynamic cell behaviour, we visualised human primary cells using an adenovirus expressing EGFP^4,5,43^. EGFP expression in virally-infected slices could be imaged repeatedly in an array, and high content 4D time-lapse of images with high resolution were generated (Fig. 5a, b). Culturing on the 3D insert dramatically prolonged the health and lifetime of primary human slices compared with those grown on a commercially available insert, as shown by less cell death and slice thinning (Supplementary Fig. 4). The improved health enabled long-term tracking of cell movement and division within a slice for up to 20 days post-infection (Supplementary Movie 4), a timescale that makes it feasible to study critical processes that occur over long time intervals such as neuronal migration. The larger slice area, substantial increase in viral-labelled cells within the human slice compared to the mouse hippocampal slice, and extended time scale necessitated automated processing and analysis of acquired data sets for single-cell analyses. To quantify processes occurring among many cells imaged over the time course at a single cell level, automated single cell tracking and segmentation was implemented. Maximum projections in *z* were taken at each time point and individual cells were segmented by automated normalised thresholding. The cells were then automatically tracked over time using a custom-written proximity-based tracking algorithm over successive frames of a 2D maximum projection (Fig. 5c). Within a typical single slice, the number of cells segmented and tracked gradually increases over time, reflecting cell divisions and new areas of viral infection (Fig. 5d). A total of 719 individual cells were tracked, and their morphological features and movement velocities were quantified over time throughout the entire slice (Fig. 5c, d). Over the course of 19 days of imaging, the size of cells initially increased rapidly, before gradually levelling off, reflecting a trend of an initial change to a condensed, elongated morphology, before pronounced thinning and expansion of processes (Fig. 5d, e). Additionally, the mean distance cells travelled gradually decreased over the time course of longitudinal imaging (Fig. 5d). From the automated quantification of distance moved on a single cell level, a subregion of significantly abnormal cell movement around 200 hpi was identified (Fig. 5c insets, Fig. 5d) and a 4D reconstruction of the area was created (Fig. 5e). This region contained outer radial glial (oRG) cells, which could be identified by their distinctive morphology that consists of a basal process extending towards the pia, while lacking direct contacts with the lateral ventricles^4^. When following single oRG cells over a series of snapshots taken once every 24 h, we observed them undergoing somal movements in the direction of the basal fibre. This somal movement was followed by the formation of a cleavage plane, the appearance of newly labelled cells after an apparent cell division, and then the return of the somata to the original location (Fig. 5e). Our sampling rate was not rapid enough to resolve cell divisions, however a similar phenomenon of somal movement of oRG cells on a time scale of minutes has been previously described in neocortical slices and termed mitotic somal translocation^4^. With our improved imaging and culture conditions, we could extend imaging of the same cells in 4D to 456 h post-infection (hpi), which allowed tracking of migrating cells that frequently moved along the basal process of the oRG (Fig. 5e, Supplementary Movie 5). Thus, with our platform, we were able to image human neocortical development in organotypic slices over longer periods than previously possible, allowing us a unique glimpse into human neocortical development.

### Single cell longitudinal changes in 4D modelling of HD slice culture

Neurodegenerative disease pathology often occurs over a long time course, and substantially precedes the appearance of clinical symptoms. Modelling and monitoring these lengthy intervals is challenging. Our platform is uniquely suited to studying neuronal phenotypes over long intervals, providing the possibility of monitoring the full course of neurodegeneration-associated phenotypes.

**Fig. 6:**
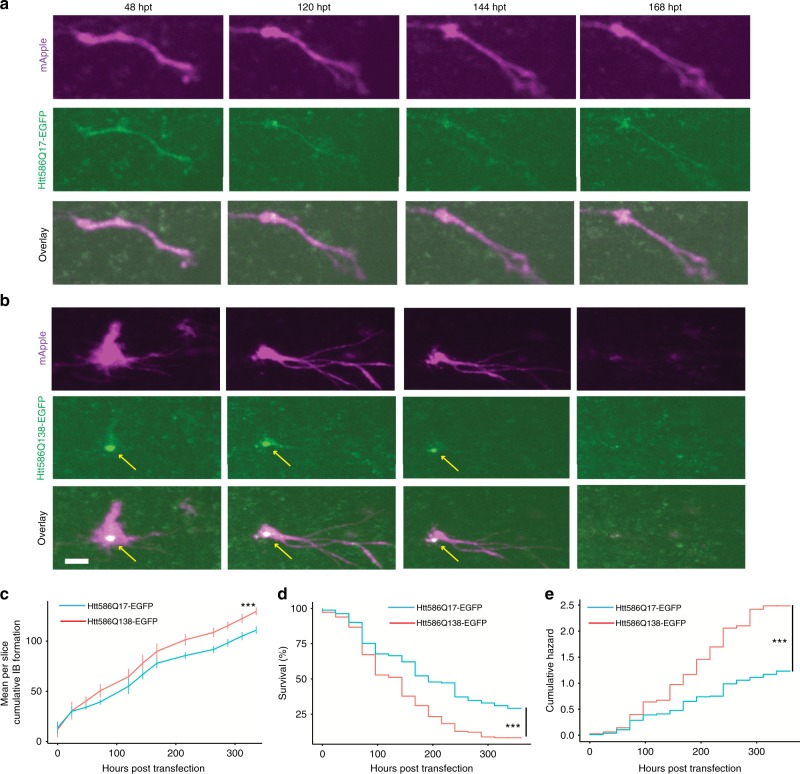
Longitudinal imaging of HTT expressing neurons in organotypic slice culture. **a**, **b** Longitudinal imaging of single neurons from hippocampal slices co-transfected with the morphology marker mApple (top) and HTT586Q17-EGFP (**a** middle) or HTT586Q138-EGFP (**b** middle) showing morphological changes and death over the course of 168 h. Yellow arrows indicate putative inclusion body formation of HTT586Q138-EGFP. HTT586Q138-EGFP transfected neuron is dead at 168 hpt. **c** Plot of mean cumulative IB formation per slice over time, showing more small particles <80 μm^2^ accumulate by 336 hpt in slices transfected with HTT586Q138-EGFP (*T*-test ***p < 0.001) and accumulation proceeds at a faster rate (ANCOVA *p* < 0.05). **d** Neurons in slices transfected with HTT586Q17-EGFP survived longer than those transfected with HTT586Q138-EGFP. **e** Plot of the cumulative risk of survival of HTT586Q17-EGFP and HTT586Q138-EGFP showing linear increases in neuronal death over time. The hazard ratio of HTT586Q138-EGFP was 1.9 in comparison to httQ17-EGFP, indicating significantly increased toxicity of HTT586Q138-EGFP (Cox proportional hazard ***p < 0.001, 95% CI 1.5–2.5, HTT586Q17-EGFP *n* = 161 neurons, HTT586Q138-EGFP *n* = 180 neurons). SEMs are indicated. Scale bar = 20 μm

To test whether our approach could be used to reveal novel neurodegenerative disease relevant phenotypes, we leveraged a well-characterised primary neuronal culture model for HD. HD is characterised by a polyglutamine (polyQ) expansion in the protein huntingtin (HTT), and polyQ expansions of over 36 are associated with disease^44^. Expression of a pathological polyQ expansion in HTT makes it prone to aggregate and form visible IBs. Prior work showed that the process of IB formation appears to function as a coping response, enabling neurons to survive mutant HTT longer than neurons with an equivalent amount of mutant HTT localised diffusely^1^. DNA encoding HTT586Q17-EGFP or HTT586Q138-EGFP was co-transfected with mApple as a neuronal morphology indicator at day 7 in culture. Slices were time-lapse imaged over the course of 336 h, beginning 24 h after transfection (Fig. 6a, b). Previously, IBs have been distinguished based on their enhanced fluorescence intensity and size ranging from 1 to 80 μm^21^. However, due to a spherical aberration commonly present in confocal 3D imaging^45^, which was also present in this study (Fig. 2), distinguishing IBs by intensity was not possible without the use of deconvolution. Nevertheless, punctate EGFP signal, likely corresponding to IBs, could be easily identified in the HTT586Q138-EGFP (Fig. 6b). HTT586Q138-EGFP transfected slices accumulated more small puncta < 80 μm^2^ per slice (*t*-test *p* < 0.0001, *n* = 9 HTT586Q138-EGFP, *n* = 12 HTT586Q17-EGFP) and at a faster rate (ANCOVA *p* < 0.05) than HTT586Q17-EGFP transfected slices (Fig. 6c), despite containing equivalent number of total segmented neurons, and neurons of equivalent size (Supplementary Fig. 5). Larger clusters of EGFP signal (>80 μm^2^) were more likely to appear in HTT586Q17-EGFP than HTT586Q138-EGFP transfected slices, likely corresponding to diffusely localised EGFP signal (*t*-test *p* < 0.0001, Supplementary Fig. 5). Neuronal death was scored by abrupt loss of mApple fluorescence, corresponding to a loss of membrane integrity (Fig. 6b), and these data were used to generate Kaplan–Meier (KM) survival curves (Fig. 6d). Slices transfected with HTT586Q17-EGFP survived longer than those transfected with HTT586Q138-EGFP, similar to reports of polyQ expanded HTT transfected primary cultured neurons (Gehan-Breslow-Wilcoxon *p* < 0.0001) (Fig. 6e, f)^1^.

Cell-culture models of inherited neurodegenerative diseases often display a consistent rate of death, suggesting the driving force of death is constant rather than brought on by cumulative damage over time^1,8,10,46^. However, this phenomenon has not yet been assayed in the more physiologically relevant context of brain slices that retain scavenger cells, such as microglia, that could alter the rate of death over time. Although the KM curve (Fig. 6d) drops off steeply at approximately 100 hpt before tapering off, the KM analysis is normalised to the total number of starting neurons, which can affect the shape of the curve. In contrast, a Cox proportional hazard (CPH) analysis^47,48^ assesses changes in the rate of the death by normalising to the number of neurons remaining (Fig. 6e). Thus, the linearity of the hazard curve can be used to assess the consistency in the rate of death over time. The hazard functions for neurons transfected with HTT586Q17-EGFP and HTT586Q138-EGFP were essentially linear (coxfit *Χ*^2^, *p* = 0.158), indicating that the expanded HTT586Q138-EGFP increases the risk of death tonically and approximately constantly over time. Additionally, the CPH hazard ratio of HTT586Q138-EGFP was 1.9, indicating the risk of death of HTT586Q138-EGFP was increased 1.9-fold compared to HTT586Q17-EGFP (*p* < 0.0001). Significant deviation (*p* < 0.05) of Cox proportional hazard did not occur between neurons transfected with HTT586Q17-EGFP and HTT586Q138-EGFP until 96 h and 5 time points of imaging, underscoring the importance of our longitudinal single-cell imaging approach. These data demonstrate our ability to do longitudinal single-cell 4D imaging of neurodegeneration, extending a powerful high throughput screening system that was first developed for monolayer primary cell cultures into a live brain tissue system with more preserved anatomy and physiology. This platform thereby broadens the biology of neurodegeneration that can be studied and increases the physiological relevance of the observations and results that emerge.

## Discussion

We designed a platform to enable longitudinal imaging of individual cells within OSC, and integrated scalable microscopic solutions to image single cells in primary tissues of developing human brain and a mouse model of neurodegeneration in 4D. The use of OSC for longitudinal imaging provides a more in vivo-like milieu in which non-cell-autonomous effects of neurodegeneration can be studied. Additionally, OSC can often be maintained for many weeks^49^, extending the timeline to study the progression of a neurodegenerative disease, and increasing the ability to mature and age the neurons. Furthermore, as longitudinal analysis of individual neurons is particularly sensitive at disambiguating the cause and effect of neuronal phenotypes towards neurodegeneration^1,8,10,27,41,50,51^, and in resolving cell lineages during neurogenesis in the brain^4,5,43^, we believe this platform will lead to new understandings of the mechanisms of neurodegeneration and neurodevelopment.

Our studies in primary rodent slice culture revealed dynamic changes in morphology and movement of some individual neurons (Fig. 4c). Neurite reorganisation was clearly observed in all of our longitudinal imaging (Figs. 2–5), and is commonly associated with neuroplasticity^52^. However, movements of neuronal somata after the brain has formed are a less commonly recognised mechanism of neuroplasticity. The cause of this neuronal movement within the slice could be due to a variety of factors, such as lingering effects of the dissection, stress from culturing conditions or biolistic transfection, or continuing development of the brain. Much of the neuronal movement observed in Fig. 4c occurs late in the life of the culture, coinciding with onset of death of surrounding neurons, suggesting neuronal death could be a trigger for neuronal reorganisation. Intriguingly, other cell types such as microglia and glia are likely also present in these slices, though were not specifically distinguished in our system. Imaging the interplay between cell types on this platform may be possible by simply using orthogonal labels specific to other cell types.

In contrast to the relatively static nature of cells within 8–10 day-old rodent brains, an abundance of cell movement was observed in GW20 primary human cortical brain slices. We were able to detect cell movement phenomena similar to mitotic somal translocations^53^ that have not been previously described. Additional phenomena such as the slow migrations of cells along the oRG scaffold were also observed, and can now be further characterised with more distinct labelling procedures and finer time resolution to confirm the cell type identity. While labelling with adeno virus in these experiments restricted labels to non-post-mitotic cells, future experiments using multiple colours and labelling strategies could be used to illuminate the complicated interplay of oRG cells and post-mitotic neurons in the development of the brain. Further studies on this interplay of oRG cells and post-mitotic neurons could also help shed light on neurological and neuropsychiatric diseases in which oRG cells have been implicated^6,54^.

Here, we present the first model of HD, to our knowledge, in which individual neurons expressing HTT586Q17-EGFP and HTT586Q138-EGFP were tracked within OSC. HD prominently affects the basal ganglia and cortex^55^, yet also can cause neuronal loss and IB formation in hippocampus^56,57^, and our model confirms that inclusion bodies occur in hippocampal slices expressing long polyQ expansions. Furthermore, we show that overexpression of HTT586Q138-EGFP is more toxic than HTT586Q17-EGFP. This provides evidence that our platform can be used as a versatile, rapid and promising model to study dominantly-inherited neurodegenerative diseases including ALS (TDP43)^10^ and PD (α-synuclein and LRRK2)^7,8^.

Due to the vast library of transgenic and mutant mouse neurodegenerative disease models that can be adapted to OSC, we believe 4D ALI can be applied to studies of many other disease models in more physiological environments. With the advent of OSC from adult neurodegenerative disease models^32,58^, our approach may also be adapted to studying neurodegeneration in aged animals. Other models of neurodegenerative disease, such as human iPS organoids, may also be well suited for this 4D ALI system. Human induced pluripotent stem cells (iPSC) have been used to generate numerous 2D models of neurodegeneration directly from human patients^59^ and have recently been applied to 3D culture by making organoids from iPSCs derived from patients^60^. While iPSC models have the advantage of not using overexpression and being fully human, they are currently much more expensive and time consuming than other cell-based models, and have difficulty fully recapitulating the full complexity of cell types, synaptic complexity, and maturity of the slice model. In contrast, use of human primary tissue provides the full complexity of cell types within the immature human brain, though it also lacks the mature synaptic complexity of an adult human brain. Overall, a complementary approach using human slice, iPSC and rodent slice culture models with 4D ALI to study underlying neuronal mechanisms will likely serve as a powerful tool in the ongoing study of neurodegenerative and developmental diseases.

One of the main advantages of this 4D microscopy platform is its versatility. For example, the ever-expanding library genetically encoded biosensors^61^ could be assayed in parallel with single cell analysis of neuronal death over time to generate an integrated model of covariates of neuronal death^8^. The sampling rate of the microscope can be increased within a focused area to handle real-time sensors of neuronal activity such as genetically encoded Ca^2+^ indicators^62^ or voltage indicators^63^. Due to the intact neurocircuitry within an organotypic slice^64^, longitudinal studies of single neurons with coordinated network activity within the slice could be performed. With the rapid acquisition speed and automated nature of this microscope platform, each of these imaging paradigms can be scaled up for high-throughput screens of neurons within organotypic slices, providing a unique platform for testing genetic modifiers, small molecules, or other perturbagens.

There is a huge and growing demand for drug therapies for neurodegenerative disease, and yet our understanding of the precise mechanisms underlying neurodegeneration has lagged. With the use of the 4D ALI platform presented here, we can begin to contribute to a greater understanding of the cause and effects of neurodegenerative disease progression and pave the way to generate new treatments and therapies to target the underlying mechanisms.

## Methods

### Animals and slice culture

Hippocampal slice cultures were prepared from 7–10 day old C57BL/6J mouse pups. 4D reconstruction was generated from a 10-day-old B6;129S-Prkd1tm1Eno/J (Jackson Laboratory) mouse. All animal experiments were approved by UCSF’s Institutional Animal and Care Use Committee (IACUC). Slice culture prep was modified from Stoppini et al.^24^ In brief, hippocampi were isolated from mouse brains and sliced using a Siskiyou tissue slicer (MS-TX) in dissection medium (100 ml MEN, 1 ml penicillin/streptomycin solution, 120 mg Tris-(hydroxymethyl)aminomethane (final concentration: 10 mM), filter-sterilised through a 0.22 µm membrane. For the Siskiyou slicer, the 400-µM winder was used make tungsten wire wrapped frames that cut through the tissue. Slices were transferred to cell culture inserts with slice culture medium (50% MEM, 25% heat-inactivated horse serum, 25% HBSS, 1% penicillin/streptomycin solution, 1 mM glucose, 1 mg/ml insulin, 0.0025% ascorbic acid, filter-sterilised through a 0.22-µm membrane), and kept in a cell culture incubator at 34 °C, 95% air, 5% CO_2_.

### Plasmids and biolistic transfection

Expression plasmids encoding the first 586 amino acids of HTT with either Q17 or Q138 fused to EGFP were cloned into the pGW1-CMV plasmid (Htt586Q17 promoter (HTT586Q17 or HTT586Q138) as previously described^65^. The Plasmid constructions were confirmed by DNA sequencing.

Preparation of plasmid microcarriers was adapted and modified using 1 µm diameter gold particles^66^. Briefly, 50 µl of 0.05 M spermidine was added to 10 mg of gold particle, placed in a sonicator for 30 s to 1 min, and then vortexed 3 times. DNA (10 µl at 1–5 mg/ml) was added to the spermidine-gold mix and briefly vortexed, and 50 µl of 1 M CaCl_2_ was added in a drop-wise fashion over a low vortex speed, and the solution was incubated at room temperature for 10 min while briefly vortexing every 60 s. The supernatant was removed after centrifugation (1000 × *g* for 1 min), and the gold pellet re-suspended in 3.5 ml of 0.075 M polyvinylpyrrollidone. The suspension was inserted into Tefzel tubing using a syringe, and the tubing placed in the tubing preparation station where the gold particles were allowed to settle before the supernatant was removed with a syringe. Next, the tubing was rotated to ensure an even spread of gold particles, which were subsequently dried under nitrogen flow. To create bullets, the tubing was cut using a tubing cutter into 1 cm length. Bullets were stored desiccated at 4 °C, and warmed to room temperature for an hour prior to use. Brain slices were transfected with biolistic gene gun (Bio-Rad) using a gas pressure of 80 psi at a distance of 10 mm. DNA-gold particle coated bullets were loaded into the cartridge of the gene gun and a modified gene gun barrel^67^ was used to transfect slices. After transfection, slices were incubated in culture media overnight.

### 3D Printed array insert and culture conditions

Custom-designed 15-well and 70-well inserts were printed using ABS material on a Lulzbot TAZ 5 printer and a custom template (see Supplementary Information). Inserts are 116 × 67 × 9 mm with troughs built at the long ends of the insert to hold approximately 3 ml of medium. Confetti of PTFE membrane (Millipore Sigma, 0.4-µm pore size, cat no. BGCM00010, 5 mm × 5 mm pieces, sterilised under ultraviolet light for an hour) was sandwiched between the plate bottom and the printed insert. At 24 h after transfection, slices were transferred in culture medium, by relocating the confetti, onto membrane within the printed array. The membrane loops around and into the troughs. The inserts were custom made to fit inside of NUNC Omni tray (Catalogue number O0764). The media in the troughs of the insert gradually decreases as it soaks through the membrane and evaporates. The tissue is fed every 1–2 days by adding fresh slice medium in the troughs to refresh the volume. Overfilling the trough can saturate the membrane and cause the slice to float and 500-700 µl fresh media was empirically determined to be the optimal addition of media every 1–2 days for rodent slices. All slices were inspected under an inverted microscope for health before imaging.

### Robotic microscope imaging system

The imaging system was built with a Nikon Ti-E base with an Applied Scientific Instruments Stage (MS-2500 and PZ-2300 with piezo Z-stage top-plate), a spinning disk confocal (Yokogawa CSU-W1), two Andor Zyla4.2 sCMOS cameras, LMM5 (Spectral) laser launch, and a Perfect Focus System (PFS) (Nikon). Imaging was performed using Nikon Plan Apo 10 × 0.5 NA and Nikon S Plan Fluor 20 × 0.45 NA objectives for large FOV and quality at depth, respectively. Illumination was provided by a Spectral LMM5 laser launch with 6 laser lines (405, 447, 488, 514, 561 and 642 nm). The system is controlled with a custom plugin for Micro-manager written in Java. Full automation of the system including the microscope, robotic arm (PAA KiNEDx) and automated incubator (LiCONiC STX-44ICBT) is achieved through the coordination of all the table elements through Green Button Go (Biosero, Fremont, CA). All hardware components of the system are commercially available, and integration was performed with some advice from respective manufacturers.

### Primary human brain slices

De-identified human tissue samples were collected with prior patient consent, in observance of the protocols approved by the Human Gamete, Embryo and Stem Cell Research Committee (institutional review board) at the University of California, San Francisco. GW20 cortical tissue was embedded in 3% low-melting agarose and sectioned at 300 μm thickness perpendicular to the ventricular plane using Leica vibratome in ice-cold artificial cerebrospinal fluid containing 125 mM NaCl, 2.5 mM KCl, 1 mM MgCl_2_, 2 mM CaCl_2_, 1.25 mM NaH_2_PO_4_, 25 mM NaHCO_3_ and 25 mM d-glucose bubbled with carbogen. Slices were transferred to commercial Millicell cell culture inserts (PICM03050) on top of PFTE (BGCM00010) membrane confetti on a six-well plate (Corning). Slices were cultured in slice medium containing 66% (vol/vol) Eagle’s basal medium, 25% (vol/vol) HBSS, 5% (vol/vol) FBS, 1% N2 supplement (ThermoFisher, #17502048), 1% Glutamax (ThermoFisher, #35050061), and 1% penicillin/streptomycin at 37 °C incubator in 5% CO_2_, 8% O_2_ at the liquid-air interface created by the slice culture insert. 24 h after dissection, slices were infected with Ad-CMV-eGFP (Vector Biolabs, #1060; 1:10,000 final dilution; MOI < 1) by dispensing 600 μl of the 2× virus inoculum. Forty-eight hours later the slice confetti were relocated to the custom 3D printed 15-well insert for time-lapse imaging. The slices were maintained in a tri-gas incubator (Sanyo) at 37 °C, 5% CO_2_, 8% O_2_ and only transferred to 37 °C, 5% CO_2_ during imaging sessions. Consecutive imaging was performed for variable intervals as indicated. Human slices were fed once a day.

### Image processing and particle analysis

Captured images were maximally projected in the *Z* dimension, montaged temporally into single files per well, and individual cells were segmented by size and pixel intensity, tracked and aligned from the same microscope field over time. Cells were tracked over time by labelling an object as the same object at the next time point based on the proximity of the coordinates its segmented mask to a segmented mask at the previous time point. The maximum proximity a cell could move was set at 64.5 μm, which was empirically determined to be further than a cell was observed to travel over the course of 24 h. Cell tracks are assigned based on smallest Euclidean distance between compared cells in two images. To track individual objects across multiple time points, we initialise each object at the first time point with a unique cell ID. For each subsequent time point, we iterate over each object in that image and assign an ID based on the closest object at the previous time point. The algorithm requires a distance cutoff (determined to be 64.5 μm in this case) that defines the furthest an individual cell can travel between sampled time points. This distance parameter would require that any cell outside the defined region have a distinct ID. More specifically, when comparing two cell objects across images taken at different time points, an initial closest distance variable, is set to the longest dimension of the image itself. The closest distance variable is updated each time a closer object is found. Once the closest object is identified, the cutoff condition is evaluated. When the closest distance variable is smaller than the defined cutoff, the object at the second time point is assigned to the same ID as the cell in the previous time point. Alternatively, when the closest distance variable is larger than the defined cutoff, the object at the second time point is further than a cell can travel between time points and the cell at the second time point receives a distinct ID. The Euclidean distance is evaluated for all measures of cell–cell separation. For analysis within the *Z* dimension, images were compiled into 4D hyperstack composite files containing aligned Z, time, and colour dimensions. For particle analysis, neurons were first segmented using maximum projected morphology channel signal at each time point to generate neuron masks within the slice. Neuron masks were generated from the mApple morphology channel and applied to the HTT-EGFP channel images to filter out signal outside neuron. Filtered images were converted to 8-bit, thresholded (5255), and particles analysed using FIJI (size = 10-infinity) to identify particles within neurons of each slice at each time point. Time of death was manually scored for each tracked neurons and used for survival analysis with time and HTT586 Q repeats as the only covariates.

### Reporting summary

Further information on research design is available in the Nature Research Reporting Summary linked to this article.

## Supplementary information

Supplementary Information

Description of Additional Supplementary Files

Supplementary Data 1

Supplementary Data 2

Supplementary Data 3

Supplementary Data 4

Supplementary Data 5

Supplementary Data 6

Reporting Summary

Supplementary Movie 1

Supplementary Movie 2

Supplementary Movie 3

Supplementary Movie 4

Supplementary Movie 5

## Data Availability

The data that support the findings of this study are available from the corresponding author upon reasonable request.

## Code availability

The code is copyright protected, and its use in performing the described research is patented (U.S. Patent 7,139,415 and U.S. Patent Application 14/737,325). Code is available upon request to the corresponding author. Access to and use of the code is subject to a non-exclusive, revocable, non-transferable, and limited right to use the code for the exclusive purpose of undertaking academic, governmental, or not-for- profit research. Use of the code or any part thereof for commercial or clinical purposes is strictly prohibited in the absence of a Commercial License Agreement from The J. David Gladstone Institutes.
