# Supplementary material for: Automated four-dimensional long term imaging enables single cell tracking within organotypic brain slices to study neurodevelopment and degeneration
Source: Commun Biol. 2019 May 1;2:155. doi: 10.1038/s42003-019-0411-9 (PMC6494885; doi:10.1038/s42003-019-0411-9)
Supplement: Supplementary file 1 — Supplementary Information [file 42003_2019_411_MOESM1_ESM.pdf]

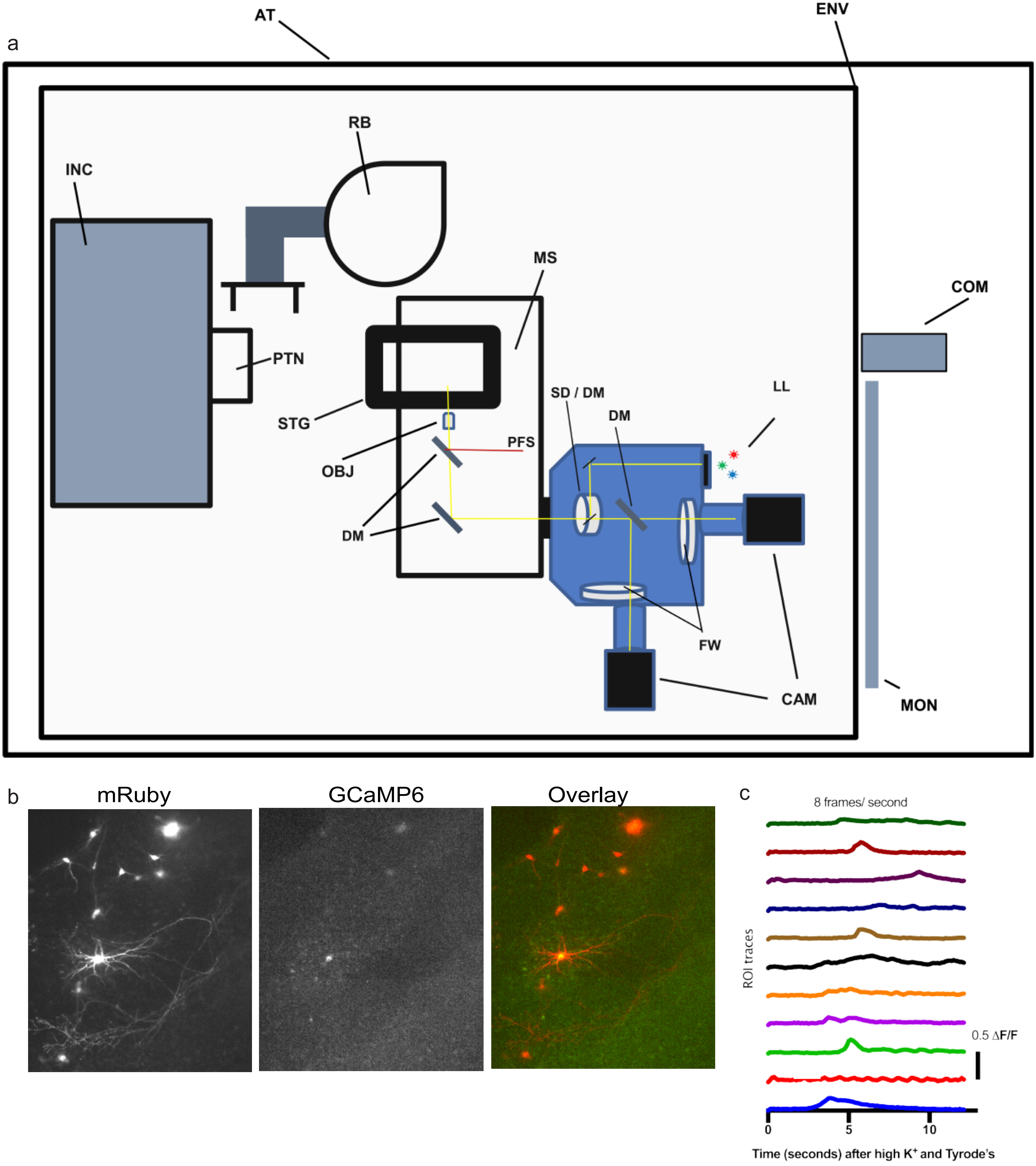
a
AT
ENV
RB
INC
MS
COM
PTN
SD / DM
LL
STG
DM
OBJ
PFS
DM
FW
CAM
MON
b
mRuby
GCaMP6
Overlay
c
8 frames/ second
ROI traces
0.5 ΔF/F
0
5
10
Time (seconds) after high K+ and Tyrode's

Supplementary Figure 1. Components and capabilities of the 4D automated imaging platform. (A) Schematic of microscope platform. Abbreviations: AT = air table, ENV = custom environmental chamber (Oko Labs), INC= automated incubator (LiCONiC), RB = automated robotic arm (PAA KiNEDx), PTN = imaging plate nest, STG= automated stage (Applied Scientific Instrumentation, MS-2500-Ti and PZ-2300), OBJ= objective, DM= dichroic mirror, SD= Spinning Disk, FD= filter wheel, LL= laser launch (Spectral), CAM= Andor Zyla 4.2 sCMOS camers, MON= monitor, COM= control computer. Image not drawn to scale. (B) A rodent hippocampal slice was transfected with hSyn1:mRuby-P2a-GCaMP6s and rapidly imaged at 8 frames per second after stimulation with addition of high K^+ and Tyrode's solution to the media. (C) Traces of mean GCaMP6s fluorescence within individual neurons in the slice after stimulation over time.

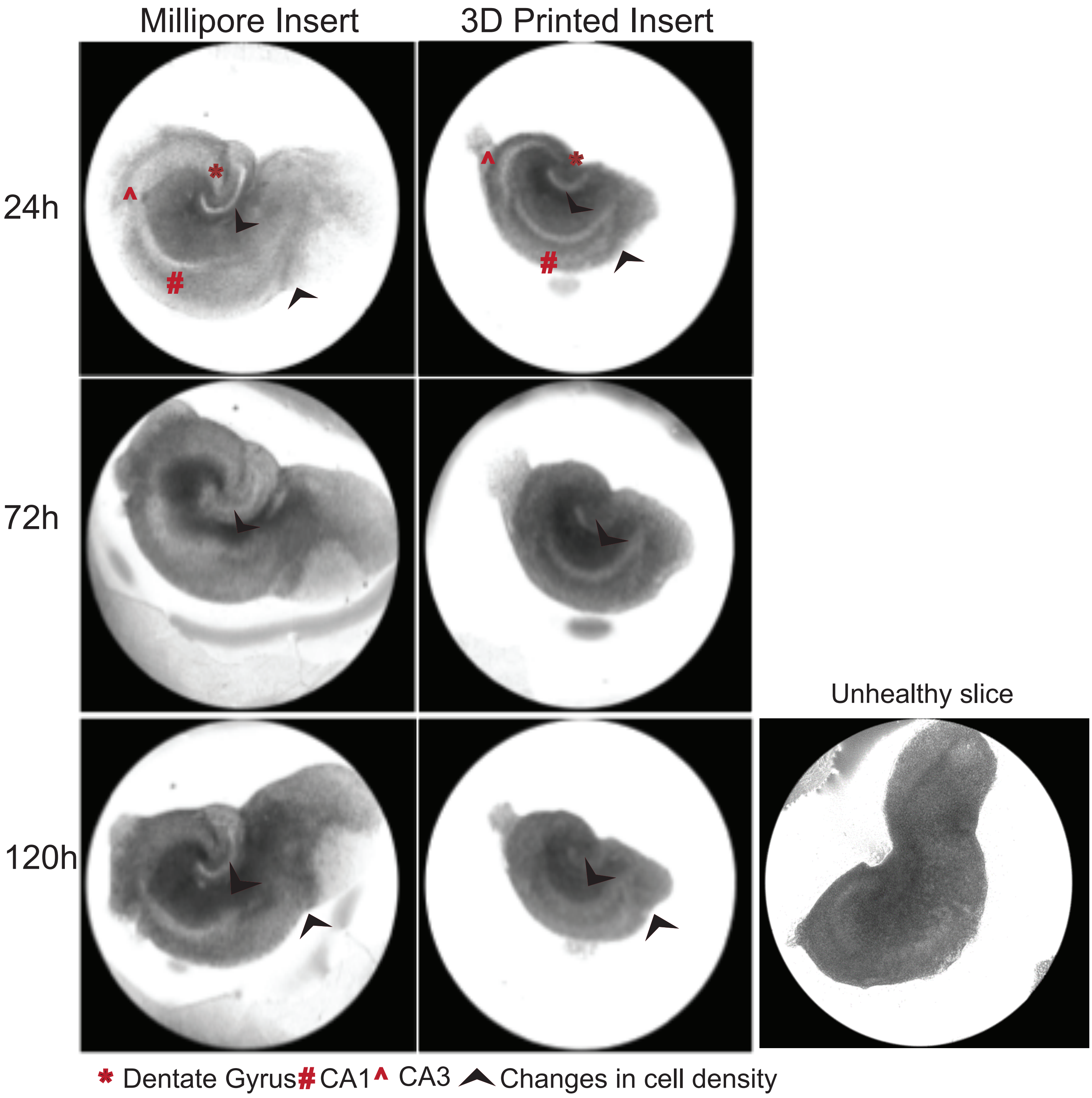
Millipore Insert
3D Printed Insert
24h
72h
120h
Unhealthy slice
* Dentate Gyrus
# CA1
^ CA3
Changes in cell density

Supplementary Figure 2. Organotypic slice cultures are healthy when maintained on 3D printed inserts. Gross morphological health of hippocampal slices over 120 hours on Millipore inserts (left) and 3D printed inserts (right). Similar increases in cell density indicative of slice health (black arrowheads), are observed in both the Millipore insert and 3D printed insert over time. Neuroanatomical features of dentate gyrus (red asterisks), and hippocampal layers CA1 (red hashtag and CA3 (red arrowhead) are clearly visible in each both slice preparations. An unhealthy slice (bottom right) lacks clear neuroanatomical features. Slices on both types of inserts show macroscopic flattening, especially visible around the edges of the slice. Over 72 hours, changes in cell density can be observed on both the commercially available and 3D printed culture setup, indicative of re-organization of cells and their processes after slicing and healthy culture. Images acquired on a Revolve microscope with a 4x objective.

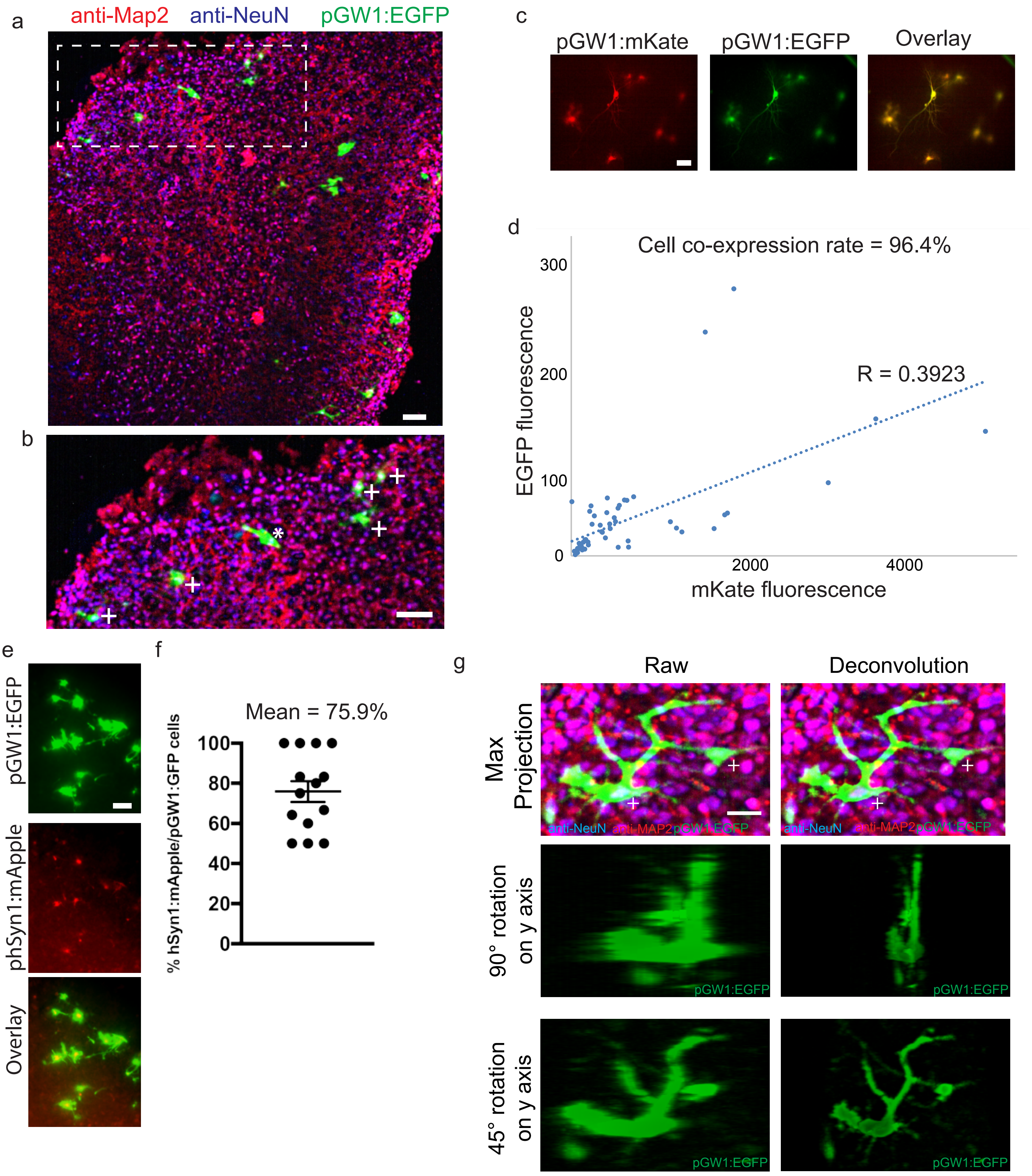

a
anti-Map2
anti-NeuN
pGW1:EGFP
b
c
pGW1:mKate
pGW1:EGFP
Overlay
d
Cell co-expression rate = 96.4%
EGFP fluorescence
mKate fluorescence
R = 0.3923
300
200
100
0
2000
4000
e
pGW1:EGFP
phSyn1:mApple
Overlay
f
Mean = 75.9%
% hSyn1:mApple/pGW1:GFP cells
100
80
60
40
20
0
g
Raw
Deconvolution
Max Projection
90° rotation on y axis
45° rotation on y axis
anti-NeuN
anti-MAP2
pGW1:EGFP

Supplementary Figure 3. Biolistic co-transfection rates and deconvolution of zstack images. (A) Slice transfected with pGW1:EGFP, fixed and counter-stained with neuron-specific anti-MAP2 and neuron-specific anti-NeuN. Scale= 75 μm. (B) Zoom in of box in (A). + denotes EGFP labelled cells that appear to be MAP2 and NeuN positive, * denotes negative. Scale= 75 μm. (C) Slice co-transfected with equal concentrations of pGW1:EGFP and pGW1:mKate shows a high rate of co-expression. Scale= 75 μm. (D) EGFP expression plotted against mKate expression from individual cells shows correlated expression (R^2= 0.3923) after co-transfection. Co-expression rate of cells was quantified as the number of cells in which both GFP and mKate signal were > 2 % above background fluorescence over the total number of cells in which at least one label was above background fluorescence. (E) A slice co-transfected with equal concentrations of pGW1:EGFP and neuron specific phSyn1:mApple shows a high rate of co-expression. Scale= 75 μm. (F) Quantification of the mean co-expression percentage of pGW1:EGFP and hSyn1:mApple across 14 slice views from 4 transfected slices, showing that most (mean = 75.9 %) pGW1:GFP expressing cells are neurons. (G) Raw (left) and computationally deconvolved (right) images of pGW1-EGFP (green) transfected neuron within an image, showing dendritic tree. (Top) overlay of pGW1-EGFP with anti-Neun (blue) and anti-MAP2 (red) identifying labelled cells (plus signs) as neurons. (Middle) 3D projection of pGW1-EGFP expression rotated 90° around the y axis. (Bottom) 3D projection rotated 45° around the y axis. Scale= 25 μm.

A

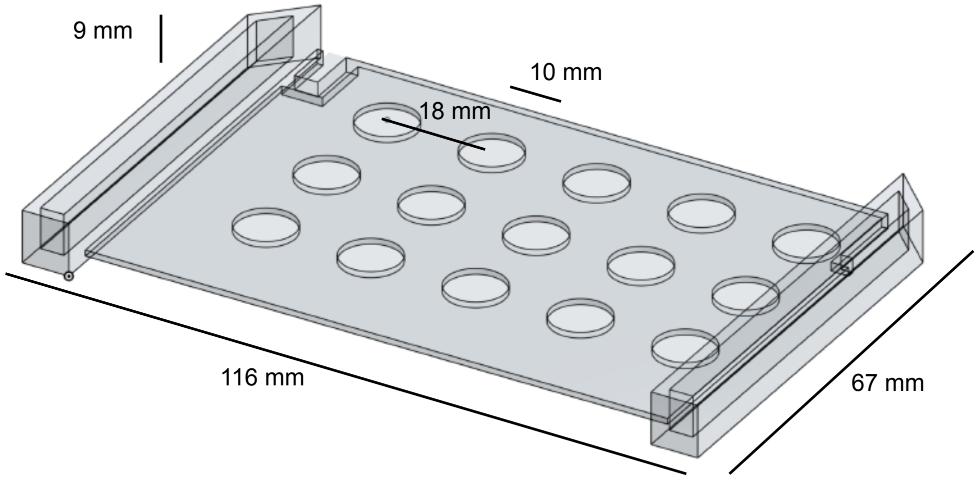
9 mm
10 mm
18 mm
116 mm
67 mm

B

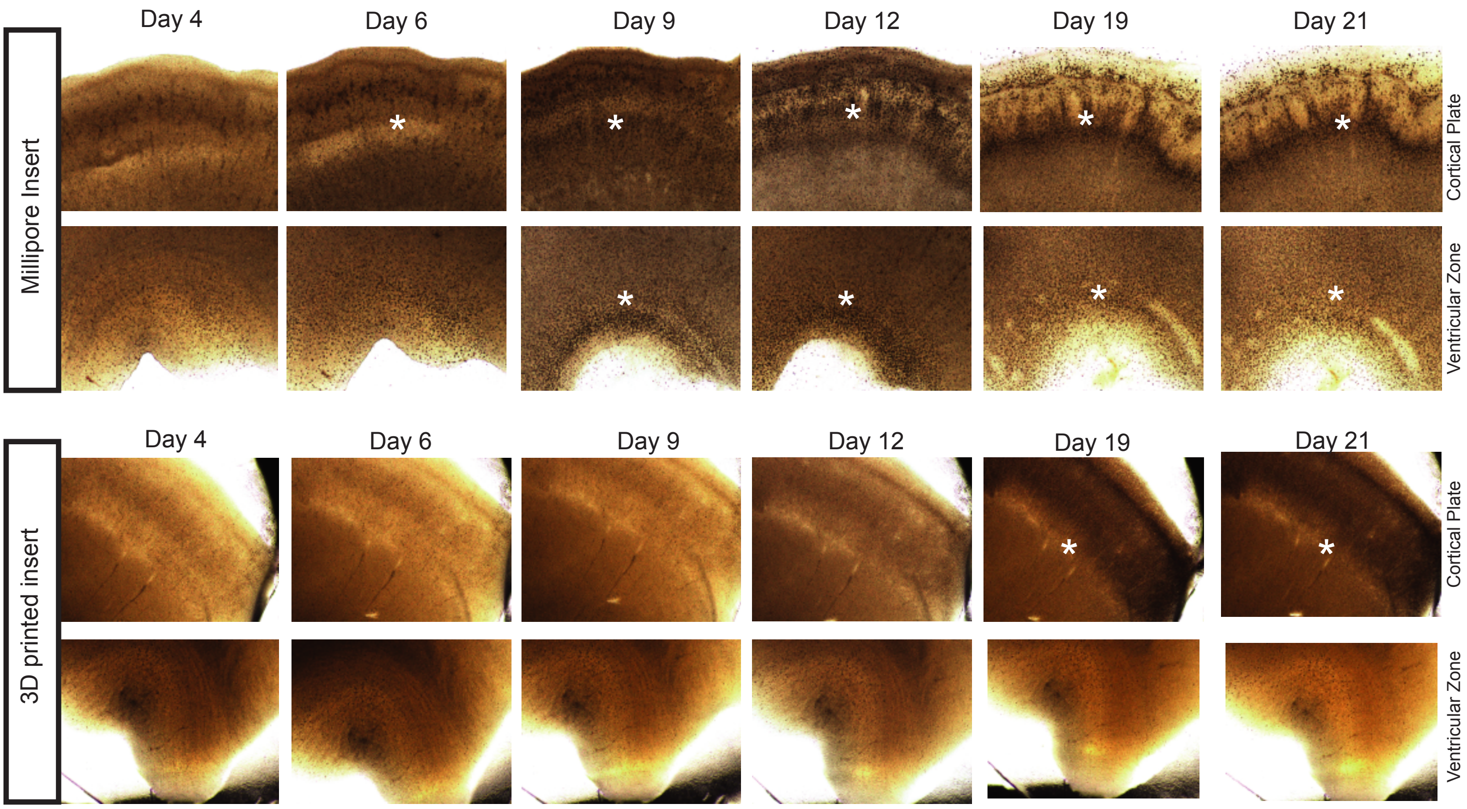
Day 4
Day 6
Day 9
Day 12
Day 19
Day 21
Millipore Insert
Cortical Plate
Ventricular Zone
3D printed insert
Cortical Plate
Ventricular Zone

Supplementary Figure 4. The 3D printed insert increases health and prolongs lifetime of human primary gestations week 20 slices. (A) Illustration of the design of a 3D printed, 15-well insert used for human GW20 slices. (B) Time lapse representative bright field image comparison of cortical plate and ventricular zone in GW20 slices on a commercial insert (Millipore Insert, Top) and the 3D printed insert (Bottom) at Days 4,6,9,12,19, and 21 in culture. Slices grown on commercial Millipore inserts display more darkened tissue and small black spots indicative of dead cells at earlier time points than samples on the 3D printed insert in both cortical plate and ventricular zone (white asterisks) (n=3 slices for each zone or condition). Images acquired on an Olympus CKX53 inverted microscope with a 20x objective.

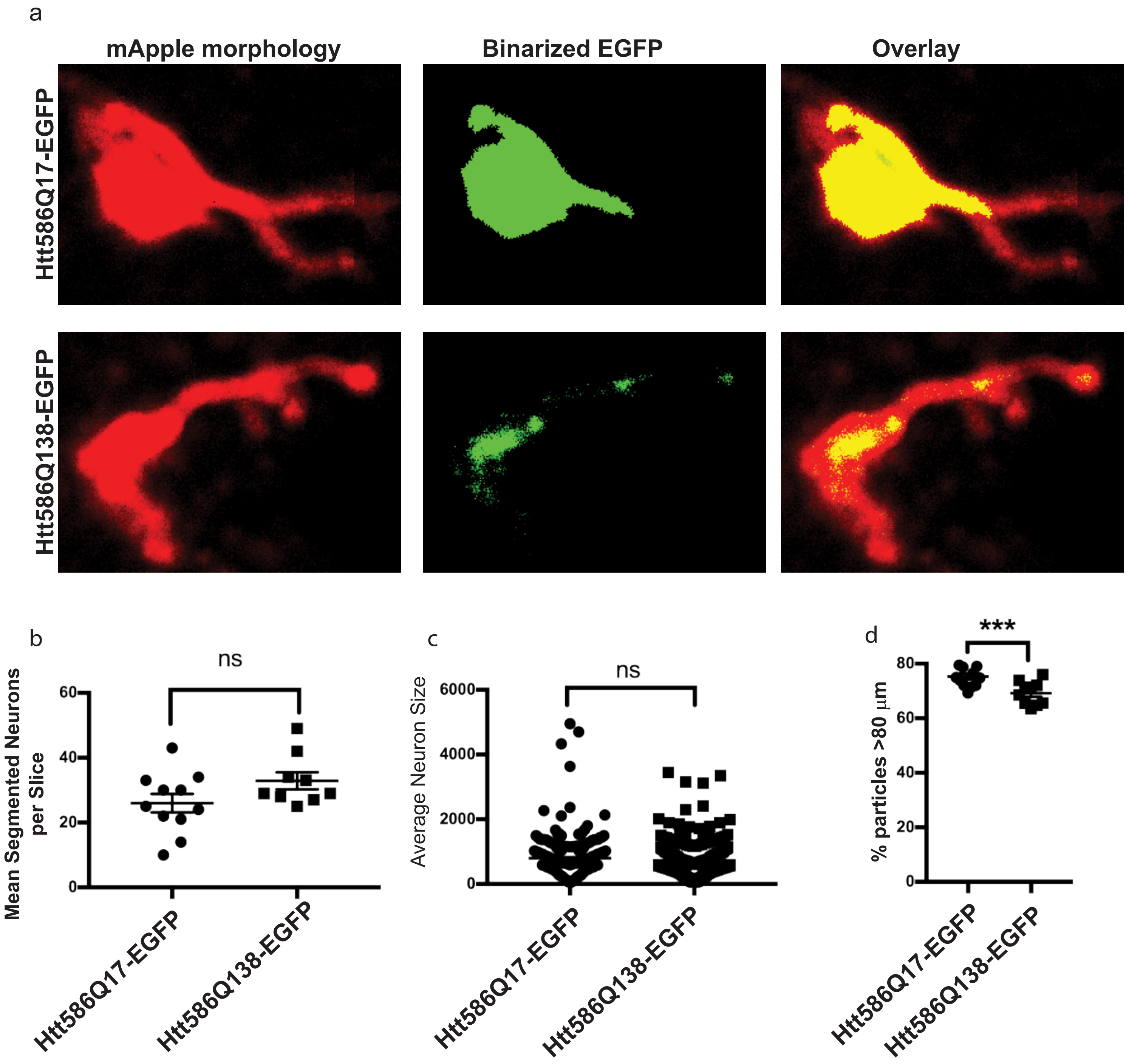
a
mApple morphology
Binarized EGFP
Overlay
Htt586Q17-EGFP
Htt586Q138-EGFP
b
ns
Mean Segmented Neurons per Slice
60
40
20
0
Htt586Q17-EGFP
Htt586Q138-EGFP
c
ns
Average Neuron Size
6000
4000
2000
0
Htt586Q17-EGFP
Htt586Q138-EGFP
d
***
% particles >80 μm
80
60
40
20
0
Htt586Q17-EGFP
Htt586Q138-EGFP

Supplementary Figure 5. Htt586Q138-EGFP transfected slices contain more small particles. (A) mApple morphology signal (left) was used to mask and quantify thresholded EGFP particles (middle) within images of slices. GFP signal within slices transfected with Htt586Q17-EGFP localized throughout neurons and larger particles of GFP. GFP signal within Htt586Q138-EGFP transfected neurons was more fragmented, with smaller particles within neurons (right). (B) Mean number of segmented particles per slice showing equivalent neuron numbers per slice in Htt586Q17-EGFP and Htt586Q138-EGFP transfected slices (*t* test p=0.1). (C) Quantification of average neuron size segmented using the morphology channel and showing no difference between Htt586Q138-EGFP and Htt586Q17-EGFP transfected slices (*t* test p =0.1). SEM indicated. (D) Mean percentage of particles per slice over 80 μm showing particles in Htt586Q17-EGFP are larger than in Htt586Q138-EGFP transfected slices.

SUPPLEMENTARY METHODS

Immunohistochemistry

Immunohistochemistry on organotypic hippocampal slice cultures was performed using a previously described protocol[1] with a few modifications. In brief, all procedures from fixation to secondary antibody washes were carried out in 6-well plates with a working solution volume of 1.5 – 1.8 mL per well. 2-3 pieces of confetti, each carrying one slice were placed on Millicell cell culture inserts as described above. 1 mL of solution was added to each well below before placing the insert, following the addition of 500-800 µl of solution on top of the slices. Prior to fixation with 20% Methanol (5 min), slices were fixed for 5 minutes with PFA (4%), containing 4% sucrose. Permeabilization solution (0.5% Triton X-100 in PBS) was incubated for 16 hours over night at 4C. The slices were incubated with blocking solution (20% BSA in PBS) for a minimum of 4 hours. Primary antibodies against NeuN (MAB377, 1:100) and MAP2 (ab5392, 1:500), diluted in 5% PBS/PBS, were incubated for 48 hours. Primary antibodies were washed off with 20% BSA in PBS with 3-4 washes of 5-10 minutes. Secondary antibodies (Alexa Fluor 405 goat anti-mouse, 1:500; Alexa Fluor 647 goat anti-chicken, 1:500), diluted in simple PBS, were incubated for 3 hours at RT. Secondary antibodies were washed as primary antibodies but using only PBS solution. Prior to imaging, the confetti carrying the slices were carefully transferred to glass slides (facing top up) and mounted using Aqua Poly/Mount (Polysciences) and 1.5 coverslips (VWR).

Plasmids

The mKate2 sequence was synthesized (Genscript) and inserted downstream of the pGW1 promoter in the pBluescriptII backbone. The hSyn1-mApple sequence was synthesized (Genscript) and subcloned into the pBluescriptII backbone. hSyn1:mRuby-P2a-GCaMP6s was ordered from addgene (#50942)[2].

Ca^{2+} Imaging

Rodent hippocampal slices were transfected with hSyn1:mRuby-P2a-GCaMP6s. A single plane with expression was manually selected and high K^+ and Tyrodes solution

were added to the slice. GFP and RFP channels were simultaneously imaged at 8 frames per second. Individual ROIs were drawn for each neuron and the mean intensity was quantified.

3D deconvolution

3D deconvolution was performed using the Iterative Deconvolve 3D and Diffraction PSF 3D plugins within ImageJ[3] with 100 iterations.

Co-transfections

For Co-labeling experiments with pGW1-EGFP and hSyn:mApple, bullets were coated with 1.5-2.5 µg DNA (of each plasmid) per 1 mg of gold particles. The slices were transfected using a gas pressure of 180 psi at the distance enabled by the regular gene gun barrel.

SUPPLEMENTARY REFERENCES

1. Gogolla N, Galimberti I, DePaola V, Caroni P. Staining protocol for organotypic hippocampal slice cultures. *Nature protocols.* 2006;1(5):2452-2456.
2. Rose T, Jaepel J, Hubener M, Bonhoeffer T. Cell-specific restoration of stimulus preference after monocular deprivation in the visual cortex. *Science (New York, NY).* 2016;352(6291):1319-1322.
3. Dougherty RP. Extensions of DAMAS and benefits and limitations of deconvolution in beamforming. *AIAA Paper 2005-2961.* 2005.
