# Supplementary material for: Automated four-dimensional long term imaging enables single cell tracking within organotypic brain slices to study neurodevelopment and degeneration
Source: Commun Biol. 2019 May 1;2:155. doi: 10.1038/s42003-019-0411-9 (PMC6494885; doi:10.1038/s42003-019-0411-9)
Supplement: Supplementary file 2 — Description of Additional Supplementary Files [file 42003_2019_411_MOESM2_ESM.pdf]

Description of Additional Supplementary Files

File Name: Supplementary Movie 1
Description: Operation of the fully automated 4D robotic microscope. Music: https://www.bensound.com/royalty-free-music.

File Name: Supplementary Movie 2
Description: Maximum projections of time-lapse imaging of neurons within hippocampal slice culture.

File Name: Supplementary Movie 3
Description: Interplay of neurons moving within slice culture.

File Name: Supplementary Movie 4
Description: Longitudinal movement of neurons through human primary tissue over 432 hours.

File Name: Supplementary Movie 5
Description: Longitudinal tracking of an oRG cell across 432 hours with 24 hour intervals in 4 dimensions. Views in X-Y axis are from a single confocal slice (magenta), and X-Z axis (red) and Y-Z axis (green) from a 3D reconstructions. Units are in µm.

File Name: Supplementary Data 1
Description: Quantification of GFP expressed in tracked neurons.

File Name: Supplementary Data 2
Description: Features of tracked objects within primary human slice tissue over time.

File Name: Supplementary Data 3
Description: Inclusion bodies found within HTT586Q17-EGFP and HTT586Q138-EGFP transfected slices over time.

File Name: Supplementary Data 4
Description: Hand curated survival statistics from slices transfected with HTT586Q17-EGFP or HTT586Q138-EGFP.

File Name: Supplementary Data 5

Description: 3D model template for printing a 15 well plate insert compatible with most 3D printers. The insert will fit into Nunc Omni tray optical imaging plates. We print on ABS material and brush with acetone to seal the area between layers. This insert fits tissue up to 9mm diameter.

File Name: Supplementary Data 6

Description: 3D model template for printing a 70 well plate insert compatible with most 3D printers. Insert will fit into Nunc Omni tray optical imaging plates. We print on ABS material and brush with acetone to seal the area between layers. This insert size fits mouse 7-10 day old organotypic hippocampal slices.
