# Supplementary material for: Automated four-dimensional long term imaging enables single cell tracking within organotypic brain slices to study neurodevelopment and degeneration
Source: Commun Biol. 2019 May 1;2:155. doi: 10.1038/s42003-019-0411-9 (PMC6494885; doi:10.1038/s42003-019-0411-9)
Supplement: Supplementary file 9 — Reporting Summary [file 42003_2019_411_MOESM9_ESM.pdf]

nature research

Corresponding author(s): Steve Finkbeiner

Reporting Summary

Nature Research wishes to improve the reproducibility of the work that we publish. This form provides structure for consistency and transparency in reporting. For further information on Nature Research policies, see Authors & Referees and the Editorial Policy Checklist.

Statistical parameters

When statistical analyses are reported, confirm that the following items are present in the relevant location (e.g. figure legend, table legend, main text, or Methods section).

n/a	Confirmed	
☐	☒	The exact sample size (*n*) for each experimental group/condition, given as a discrete number and unit of measurement
☐	☒	An indication of whether measurements were taken from distinct samples or whether the same sample was measured repeatedly
☐	☒	The statistical test(s) used AND whether they are one- or two-sided<br>*Only common tests should be described solely by name; describe more complex techniques in the Methods section.*
☐	☒	A description of all covariates tested
☐	☒	A description of any assumptions or corrections, such as tests of normality and adjustment for multiple comparisons
☐	☒	A full description of the statistics including central tendency (e.g. means) or other basic estimates (e.g. regression coefficient) AND variation (e.g. standard deviation) or associated estimates of uncertainty (e.g. confidence intervals)
☐	☒	For null hypothesis testing, the test statistic (e.g. *F*, *t*, *r*) with confidence intervals, effect sizes, degrees of freedom and *P* value noted<br>*Give P values as exact values whenever suitable.*
☒	☐	For Bayesian analysis, information on the choice of priors and Markov chain Monte Carlo settings
☒	☐	For hierarchical and complex designs, identification of the appropriate level for tests and full reporting of outcomes
☒	☐	Estimates of effect sizes (e.g. Cohen's *d*, Pearson's *r*), indicating how they were calculated
☐	☒	Clearly defined error bars<br>*State explicitly what error bars represent (e.g. SD, SE, CI)*

Our web collection on statistics for biologists may be useful.

Software and code

Policy information about availability of computer code

Data collection	Micromanager is an open source platform for programming microscopes. Full automation of the system was performed with Green Button Go (Biosero, Freemont). The code is copyright protected, and its use in performing the described research is patented (U.S. Patent 7,139,415 and U.S. Patent Application 14/737,325). Code is available upon request to the corresponding author. Access to and use of the code is subject to a non-exclusive, revocable, non-transferable, and limited right to use the code for the exclusive purpose of undertaking academic, governmental, or not-forprofit research. Use of the code or any part thereof for commercial or clinical purposes is strictly prohibited in the absence of a Commercial License Agreement from The J. David Gladstone Institutes
Data analysis	Galaxy Project is an open source, web-based platform for computational analysis. https://galaxyproject.org/ Version 16.10 of the platform is used in this work. The infrastructure provided by the Galaxy platform is used to to run our custom code. Custom R and ImageJ scripts were also used in data analysis and their repositories were also used in analysis. No commercial software was used in the analysis.

For manuscripts utilizing custom algorithms or software that are central to the research but not yet described in published literature, software must be made available to editors/reviewers upon request. We strongly encourage code deposition in a community repository (e.g. GitHub). See the Nature Research guidelines for submitting code & software for further information.

Data

Policy information about availability of data

All manuscripts must include a data availability statement. This statement should provide the following information, where applicable:

- Accession codes, unique identifiers, or web links for publicly available datasets
- A list of figures that have associated raw data
- A description of any restrictions on data availability

Robotic imaging data sets are too large to share in an online public repository, but will be made available upon request

Field-specific reporting

Please select the best fit for your research. If you are not sure, read the appropriate sections before making your selection.

☒ Life sciences ☐ Behavioural & social sciences ☐ Ecological, evolutionary & environmental sciences

For a reference copy of the document with all sections, see nature.com/authors/policies/ReportingSummary-flat.pdf

Life sciences study design

All studies must disclose on these points even when the disclosure is negative.

Sample size	Sample size for survival analysis was determined by the amount of neurons that were biolistically transfected in a single batch without any prior assumptions for variance or effect size. Post-hoc, we calculate the sample size required to see significance for our effect size would be 94 neurons tracked for each condition, though we used over 161 neurons for this analysis. Put an alternative way, with the sample size we used, we could have determined an effect size of a hazard ratio of between 1.4 and 1.5, but we empirically found 1.9. For primary human tissue, we generated data based on the availability of samples.
Data exclusions	Parameters to distinguish IB formation were validated on held out and excluded data before being applied to the remaining data set. In initial attempts at imaging primary human tissue, we did not maintain the slices in 8% oxygen between imaging sessions, and data acquired during those attempts was excluded.
Replication	At least one other instance of similar phenomenon were observed in each case where described, suggesting these were representative events.
Randomization	Samples were randomized due to the random collection of human primary tissue specimens and were processed in random order.
Blinding	Neuronal death for survival analysis was scored on time lapse images without knowing the experimental condition (HttQ17 vs HttQ138).

Reporting for specific materials, systems and methods

Materials & experimental systems

n/a	Involved in the study
☒	☐ Unique biological materials
☒	☐ Antibodies
☒	☐ Eukaryotic cell lines
☒	☐ Palaeontology
☐	☒ Animals and other organisms
☒	☐ Human research participants

Methods

n/a	Involved in the study
☒	☐ ChIP-seq
☒	☐ Flow cytometry
☒	☐ MRI-based neuroimaging

Animals and other organisms

Policy information about studies involving animals; ARRIVE guidelines recommended for reporting animal research

Laboratory animals	7-10 day old C57BL/6J and B6;129S-Prkd1tm1Eno/J were used and were not sexed, and we expect approximately equal amounts of males and females in our experiments
Wild animals	This study did not use wild animals

Field-collected samples | This study did not use field-collected animals
